# Supplementary material for: Hepatitis C prevalence and key population size estimate updates in San Francisco: 2015 to 2019
Source: PLoS One. 2022 May 11;17(5):e0267902. doi: 10.1371/journal.pone.0267902 (PMC9094540; doi:10.1371/journal.pone.0267902)
Supplement: S2 Table — Eleven unique abstracts were retained for further review after following the literature search methods described in S1 Table. These abstracts are listed below. (DOCX) [file pone.0267902.s002.docx]

**S2 Table. Abstracts Retained for Further Review After Literature Search.**

Eleven unique abstracts were retained for further review after following the literature search methods described in Table S1. These abstracts are listed below.

|  | **First Author** | **Title** | **Journal** | **Pub Year** | **Used in Analysis?** |
| --- | --- | --- | --- | --- | --- |
| 1 | Raymond, H.F. | Estimated population size of men who have sex with men, San Francisco, 2017 | AIDS and Behavior | 2019 | yes |
| 2 | Wesson, P. | Estimating population size of transwomen in San Francisco using multiple methods, 2013. | International Journal of Transgenderism | 2017 | yes |
| 3 | Crissman, H.P. | Transgender demographics: A household probability sample of US adults, 2014. | American Journal of Public Health | 2017 | yes |
| 4 | Raymond, H.F. | Transwoman population size | American Journal of Public Health | 2017 | yes |
| 5 | Hernandez, C.J. | High hepatitis C virus seropositivity, viremia, and associated risk factors among trans women living in San Francisco, California | PloS ONE | 2021 | no, duplicate of TEACH4 dataset |
| 6 | Mirzazadeh, A. | Progress toward closing gaps in the hepatitis C virus cascade of care for people who inject drugs in San Francisco | PloS ONE | 2021 | no, duplicate of PWID NHBS 2018 dataset |
| 7 | Morris, M.D. | Treatment cascade for hepatitis C virus in young adult people who inject drugs in San Francisco: Low number treated | Drug and Alcohol Dependence | 2019 | no, duplicate of UFO dataset |
| 8 | Page, K. | HCV incidence is associated with injecting partner age and HCV serostatus mixing in young adults who inject drugs in San Francisco | PloS ONE | 2019 | No, duplicate of UFO dataset |
| 9 | Facente, S.N. | Estimated hepatitis C prevalence and key population sizes in San Francisco: A foundation for elimination | PloS ONE | 2018 | no, did not want to include estimates calculated using overlapping data in 2015 |
| 10 | Schackman, B.R. | Cost-effectiveness of hepatitis C screening and treatment linkage intervention in U.S. methadone maintenance treatment programs | Drug and Alcohol Dependence | 2018 | no, duplicate of pre-existing estimate from Perlman, *et al.* |
| 11 | Morris, M.D. | Geographic differences in temporal incidence trends of hepatitis C virus infection among people who inject drugs: The InC3 collaboration | Clinical Infectious Diseases | 2017 | No, duplicate of UFO dataset |
